# Colonic hematoma after extracorporeal shock wave lithotripsy for pancreatic stones: a case report

**DOI:** 10.1186/s12876-019-1117-7

**Published:** 2019-12-04

**Authors:** Yu Liu, Lu Hao, Teng Wang, Zhao-Shen Li, Zheng-Lei Xu, Liang-Hao Hu

**Affiliations:** 1 Department of Gastroenterology, Gongli Hospital, The Second Military Medical University, 800 Xiangyin Road, Shanghai, 200433 China; 2 Department of Gastroenterology, Changhai Hospital, The Second Military Medical University, 800 Xiangyin Road, Shanghai, 200433 China; 3 Department of Gastroenterology, First Affiliated Hospital, Zhejiang University School of Medicine, Hangzhou, China; 4 Department of Gastroenterology, The Second Clinical Medical College (Shenzhen People’s Hospital), Jinan University, 1017 North Dongmen Road, Shenzhen, Guangdong China

**Keywords:** Colonic hematoma, Extracorporeal shock wave lithotripsy, Pancreatic stones, Complication

## Abstract

**Background:**

Despite pancreatic extracorporeal shock wave lithotripsy (P-ESWL) is a minimally invasive treatment for pancreatic stones, complications exist.

**Case presentation:**

A 37-year-old male was diagnosed with chronic pancreatitis and admitted to our hospital for recurrent acute pancreatitis. After the first P-ESWL session, the patient complained of a new type of pain different from the previous pain pattern. Computerized tomography and colonoscopy were arranged and colonic hematoma was found. Since the patient had stable vital signs, no special treatment was given focusing on the colonic hematoma. Five days later, P-ESWL treatment was repeatedly performed for four consecutive days. Two days after the last P-ESWL session, the patient underwent endoscopic retrograde cholangiopancreatography. At the three-month follow up visit, the colonic hematoma disappeared and pancreatic stones decreased significantly.

**Conclusions:**

To the best of our knowledge, colonic hematoma after P-ESWL for pancreatic stones has never been reported. Here, we present the only case of colonic hematoma after P-ESWL, which was coincidentally found in more than 6000 P-ESWL sessions in our hospital. As the symptoms of colonic hematoma are mild, we believe the incidence of colonic hematoma has been underestimated. Many people with colonic hematoma after P-ESWL may be undiagnosed or misdiagnosed. Treatment for colonic hematoma depends on whether there is severe clinical state. Exploration of more precise location method for pancreatic stones may reduce the probability of P-ESWL complication.

## Background

Pancreatic stones are pathognomonic changes of chronic pancreatitis (CP). It has been reported that up to 50% of patients with CP have pancreatic duct stones at long-term follow-up [1]. Pancreatic stones may cause pancreatic duct obstruction and ductal hypertension which could contribute to abdominal pain and affect the quality of life of patients [2]. Therefore, removing pancreatic stones is of significant importance. Pancreatic stones can be treated with surgical or endoscopic techniques or by pancreatic extracorporeal shock wave lithotripsy (P-ESWL) or endoscopic techniques combining with P-ESWL. P-ESWL is a safe, effective and minimally invasive method to treat pancreatic stones which was first applied in 1987 [3]. While, some complications have been reported in the past 30 years. To the best of our knowledge, colonic hematoma caused by P-ESWL has never been reported. Here, we present the only case of colonic hematoma after P-ESWL, which was coincidentally found in more than 6000 P-ESWL sessions in our hospital.

## Case presentation

A 37-year-old male was admitted to our department due to recurrent acute pancreatitis for 11 years. Computed tomography (CT) revealed the upstream dilation of the pancreatic with the radiopaque stones in the pancreatic body. The result confirmed the diagnosis of CP.

Upon admission, blood routine test, liver and kidney routine function test and other faecal routine test were all examined and they were all within normal range. There was no contradiction of P-ESWL. P-ESWL treatment was performed on the patient by using the third-generation lithotripter (Delta Compact II, Dornier Med Tech, Wessling, Germany) with the patient in a 30°-right supine position. During each P-ESWL therapeutic session, up to 5000 shock waves were delivered at a frequency of 120 shocks/min with an intensity of 6 (16,000 kV) and a scale of 1 to 6 [4]. During the procedure, furbiprofen and remifentanil were combined for analgesia by intravenous infusion. After the first session of P-ESWL, the stones were partially pulverized.

**Fig. 1:**
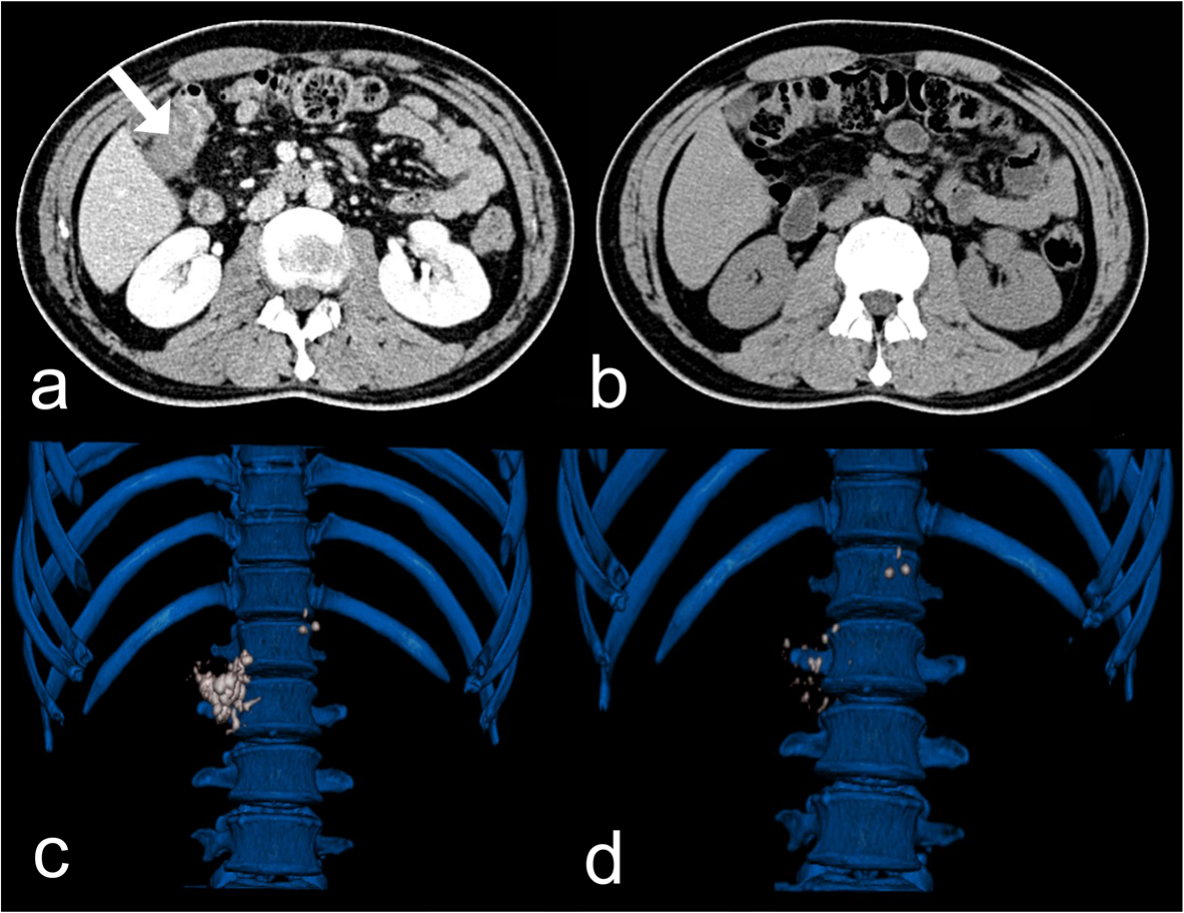
**a** The contrast-enhanced computerized tomography (CT) scan shows there is a colonic space occupying lesion (white arrow) in the hepatic flexure of colon. **b** CT scan shows the colonic hematoma disappeared at the three-month follow up. **c** The three-dimensional CT reconstruction of abdominal shows the pancreas with radiopaque stones in the whole pancreatic body before pancreatic extracorporeal shock wave lithotripsy (P-ESWL). **d** Three-dimensional reconstruction of CT scan shows the pancreatic stones has decreased significantly at the three-month follow up

**Fig. 2:**
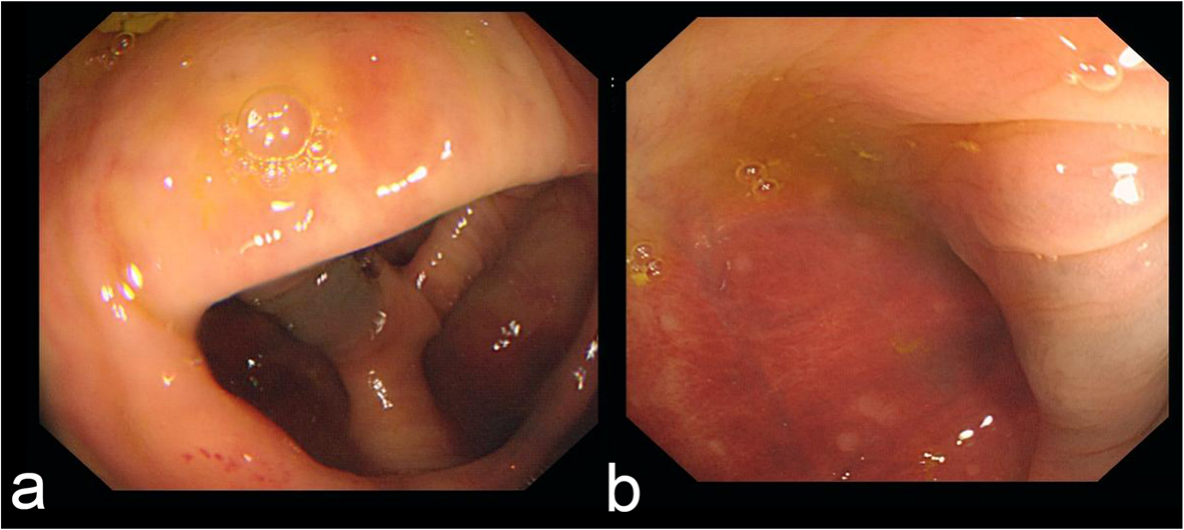
**a**, **b** The colonoscopy shows a colonic hematoma after P-ESWL. There is congestion of blood under serous of colonic wall and the mucous of colon is intact

The serum amylase after the first P-ESWL was normal. But the patient complained a slight abdominal pain which was different from the previous pain pattern. To check if there was any other new problem, a contrast-enhanced CT for upper abdomen was arranged immediately. The enhanced CT scan after P-ESWL showed there was nothing abnormal about the pancreas but an unknown colonic space occupying lesion was found in the hepatic flexure of colon (Fig. 1a). There was no sign of colonic obstruction. The patient denied any dizzy or gastrointestinal symptoms, such as nausea, vomiting, or melena. Physical examination revealed his abdomen was flat, soft, nondistended, and nontender with no hepatosplenomegaly appreciated. Therefore, colonoscopy examination was arranged on the second day after the CT scan to make further examination. The patient was given intestinal cleansing program and completed bowel preparations in the morning of the scheduled colonoscopy (by taking an intestinal cleansing solution, composed of 2 L of polyethylene glycol-3350 solution and 300 mL of magnesium citrate). During bowel cleansing, our patient did not observe any blood in his stool. Then the colonoscopy was performed. The colonoscopy result showed there was congestion of blood under the colonic mucosa while the mucosa of the colonic wall was intact at 65 cm distance from anal verge which revealed it was a colonic hematoma after P-ESWL (Fig. 2a, b). The patient was ordered to fast, lie on bed and reduce activities all day. Fluids were transfused and vital signs were monitored all day. The patient was quite stable in the following day and abdominal pain disappeared gradually. Considering that the patient had stable vital signs and no fresh bleeding was observed, no special treatment was given focusing on the colonic hematoma. After 5 days, P-ESWL was performed again on the patient. P-ESWL therapeutic sessions were performed for four consecutive days. The serum amylases after P-ESWL were all within normal range. Two days after the last P-ESWL session, endoscopic retrograde cholangiopancreatography was performed in which pancreatic duct was cleaned to extract the residual pancreatic stones. At the three-month follow up, the colonic hematoma disappeared (Fig. 1b) and pancreatic stones decreased significantly (Fig. 1c, d). At the two-year follow up, the patient had no pain attack any more.

## Discussion and conclusions

P-ESWL is a safe and effective minimally invasive method to treat pancreatic stones [1, 2]. Complications after P-ESWL contain post-P-ESWL pancreatitis, bleeding, infection, steinstrasse and perforation [4]. The mechanisms for occurrence of these complications may be as follows: on the one hand, the organs along the shock wave conduction pathway may be affected by the release of energy of the shock wave; on the other hand, the position of the pancreas changes with the respiratory motion so that the shock wave generator cannot always locate the target [5]. Among the complications, bleeding occurring in bounded organ is rarely reported with the reported incidence rate of 0.3% [4]. The previously reported hematoma includes: hepatic subcapsular hematoma [6], hilar hematoma [7] and splenic hematoma [8]. To date, colonic hematoma has not been reported before.

Colonic hematoma presents with non-specific symptoms: abdominal pain, vomiting, abdominal masses, diarrhea, and melena [9, 10]. Colonic hematoma after P-ESWL revealed even slighter symptoms because of restricted energy of shock wave and the focus of pancreas during P-ESWL. Such cases may be easily ignored and misdiagnosed because of its non-specific symptom. As a result, incidence of colonic hematoma after P-ESWL could probably be underestimated. In the present case, the patient only had slight abdominal pain without any other syptoms. We could diagnose this complication because the patient complained a new pattern of abdominal pain which was different from before. This observation underlines the importance of assessing thoroughly the patient after each P-ESWL procedure and taking into consideration every new symptoms or modification of a pre-existing (such as pain), in order to detect eventual P-ESWL-related complications.

**Fig. 3:**
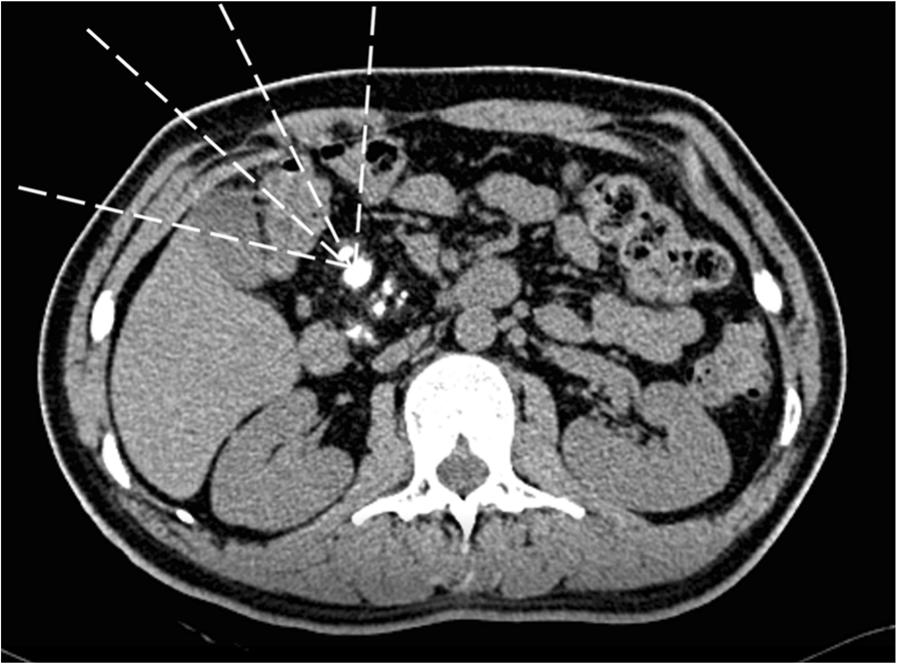
The computerized tomography (CT) shows plenty of radiopaque pancreatic stones in the pancreas. The white dashed lines indicate the shock wave. The conduction pathway of shock wave is a three-dimensional cone space and the hepatic flexure of colon is on the conduction pathway

**Fig. 4:**
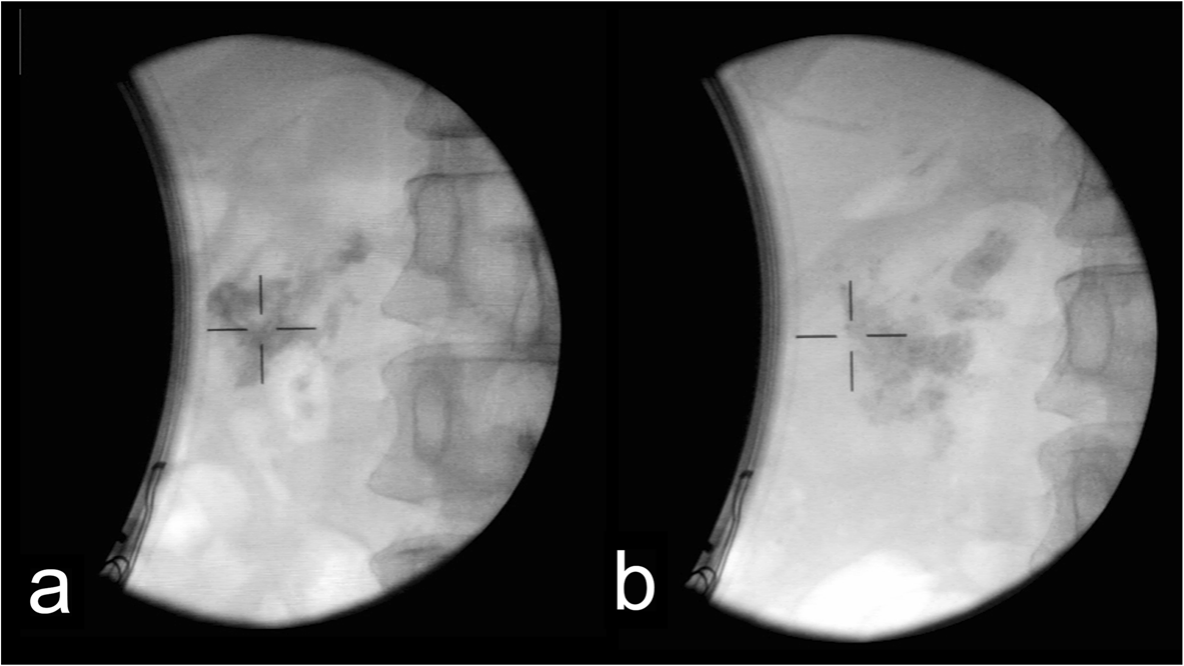
X-ray image of the extracorporeal shock wave lithotripsy was photographed in a 30°-right supine position of patients. **a** The X-ray image for pancreatic stone of the patient before the first session of P-ESWL. **b** The X-ray image for pancreatic stone of the patient after the last session of P-ESWL

Colonic hematoma often occurs due to wound and iatrogenic injury, some colonic hematoma can also occur spontaneously [9, 11–16]. In our case, colonic hematoma occurred because the hepatic flexure of colon was on the conduction pathway of shock wave and also the shock wave generator cannot locate the target continuously because the pancreas can move during respiration (Figs. 3, 4). Reported cases of colonic hematoma are most commonly treated conservatively [17–19]. In our case, the patient had no obvious sign of blood loss and the vital signs were stable. The colonoscopy showed the hematoma was limited under the colonic mucosa while the mucosa of the colonic wall was intact. No signs about bowal obstruction were found out. And abdominal pain of the patient disappeared gradually. Hence, the colonic hematom was supposed to be stable and limited. According to this, the patient was asked to fast and lie on bed. Fluids were transfused and vital signs were monitored closely. It turned out that the colonic hematoma was stable.

Colonic hematoma casued by P-ESWL is a rare clinical complication with nonspecific symptoms. Diagnosing colonic hematoma before patients present any severe clinical state (like bowel obstruction or hypotension) is of significant importance. When the condition is stable and the colonic hematoma is limited, conservative treatment could be the first choice. When there is severe clinical state, surgery may be needed to deal with bowel obstruction or hypotension.

Since injury of colon caused by shock wave is frequently limited and rarely happened, no specific change is needed to make during the ESWL procedure. Yet, method of more precise location for pancreatic stones during the ESWL procedure may be explored to reduce the dislocation of pancreatic stones due to pancreas moving when respiration. With more precise location method, probability of P-ESWL complication may be reduced.

In conclusion, complications, especially bleeding of other organs, caused by P-ESWL are usually limited due to the limited energy of shock wave and the focus of pancreas. As a result, many cases of colonic hematoma after P-ESWL for pancreatic stones were likely to be undiagnosed or misdiagnosed for patients with few and non-specific symptoms. Treatment for colonic hematoma depends on whether there is severe clinical state. Exploration of more precise location method for pancreatic stones may reduce the probability of P-ESWL complication.

## Data Availability

All data generated or analyzed during this study are included in this current article.

## Abbreviations

CP: Chronic pancreatitis
CT: Computed tomography
P-ESWL: Pancreatic extracorporeal shock wave lithotripsy
